# Genetic diversity of equine herpesvirus 1 isolated from neurological, abortigenic and respiratory disease outbreaks

**DOI:** 10.1111/tbed.12809

**Published:** 2018-02-09

**Authors:** N. A. Bryant, G. S. Wilkie, C. A. Russell, L. Compston, D. Grafham, L. Clissold, K. McLay, L. Medcalf, R. Newton, A. J. Davison, D. M. Elton

**Affiliations:** ^1^ Animal Health Trust Kentford Newmarket Suffolk UK; ^2^ MRC‐University of Glasgow Centre for Virus Research Glasgow UK; ^3^ Department of Veterinary Medicine University of Cambridge Cambridge UK; ^4^ Sheffield Children's NHS Foundation Trust Sheffield South Yorkshire UK; ^5^ Earlham Institute Norwich Research Park Norwich Norfolk UK

**Keywords:** equine herpesvirus type 1, sequencing, diversity

## Abstract

Equine herpesvirus 1 (EHV‐1) causes respiratory disease, abortion, neonatal death and neurological disease in equines and is endemic in most countries. The viral factors that influence EHV‐1 disease severity are poorly understood, and this has hampered vaccine development. However, the N752D substitution in the viral DNA polymerase catalytic subunit has been shown statistically to be associated with neurological disease. This has given rise to the term “neuropathic strain,” even though strains lacking the polymorphism have been recovered from cases of neurological disease. To broaden understanding of EHV‐1 diversity in the field, 78 EHV‐1 strains isolated over a period of 35 years were sequenced. The great majority of isolates originated from the United Kingdom and included in the collection were low passage isolates from respiratory, abortigenic and neurological outbreaks. Phylogenetic analysis of regions spanning 80% of the genome showed that up to 13 viral clades have been circulating in the United Kingdom and that most of these are continuing to circulate. Abortion isolates grouped into nine clades, and neurological isolates grouped into five. Most neurological isolates had the N752D substitution, whereas most abortion isolates did not, although three of the neurological isolates from linked outbreaks had a different polymorphism. Finally, bioinformatic analysis suggested that recombination has occurred between EHV‐1 clades, between EHV‐1 and equine herpesvirus 4, and between EHV‐1 and equine herpesvirus 8.

## 1 INTRODUCTION

Equine herpesvirus 1 (EHV‐1; species *Equid alphaherpesvirus 1*) can have a devastating effect on horses. Respiratory infections are common, and the virus can also cause multiple abortion outbreaks, neonatal death and neurological damage that may lead to fatal paralysis (Goehring, Landolt, & Morley, 2010; Irwin et al., 2007; Mumford et al., 1987; Schulman, Becker, van der Merwe, Guthrie, & Stout, 2014). After initial infection of the upper respiratory tract epithelium, a highly cell‐associated viraemia develops (Gibson, Slater, & Field, 1992; Scott, Dutta, & Myrup, 1983). EHV‐1‐infected peripheral blood mononuclear cells (PBMCs) then spread the virus to endothelial cells lining the blood vessels. If this occurs in the pregnant uterus or in the central nervous system, the resulting tissue damage can cause abortion or myeloencephalopathy (Edington, Smyth, & Griffiths, 1991; Whitwell & Blunden, 1992). Despite the potential severity of outbreaks and the financial losses incurred as a result, there are no vaccines licensed to protect against neurological disease, and outbreaks, as well as cases of abortion, still occur in highly vaccinated animals. This was recently highlighted by the abortion storm recorded in Hertfordshire (United Kingdom; UK) in 2016 in fully vaccinated animals (http://www.aht.org.uk/cms-display/interim-report16-april2.html).

**Figure 1:**
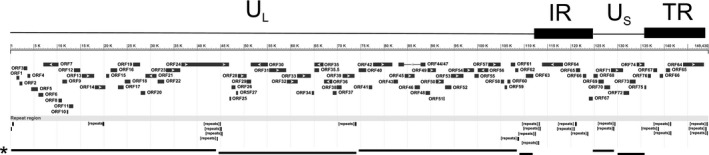
EHV‐1 genome structure showing functional ORFs, inverted repeats and tandem repeats (adapted from GenBank accession NC_001491.2). Inverted repeats are shown as black rectangles, and unique regions as black lines above the ORFs. Regions used for subsequent sequence analysis are indicated with an asterisk (*)

EHV‐1 was first isolated from abortion material from the USA in the 1930s (Dimock & Edwards, 1933). Subsequent sequenced‐based phylogenetic analysis led to the classification of the virus in the genus *Varicellovirus* (family *Herpesviridae*), together with its close relatives equine herpesvirus 4 (EHV‐4; species *Equid alphaherpesvirus 4*) and equine herpesvirus 8 (EHV‐8; species *Equid alphaherpesvirus 8*). The linear, double‐stranded genomes of these viruses share a common structure. Thus, the EHV‐1 genome is approximately 150 kbp in size and consists of long and short unique regions (U_L_ and U_S_, respectively), the former flanked by a small inverted repeat (TR_L_/IR_L_) and the latter by a large inverted repeat (TR_S_/IR_S_) (Figure 1) (Henry et al., 1981; Pellett & Roizman, 2007; Telford, Watson, McBride, & Davison, 1992; Whalley, Robertson, & Davison, 1981; Yalamanchili & O'Callaghan, 1990). The genome contains 76 open reading frames (ORFs) predicted to encode functional proteins, four of which are duplicated in TR_S_/IR_S_. The average nucleotide composition of the genome is 56.7% G+C, although it is significantly higher in TR_S_/IR_S_, at 67% (Telford et al., 1992).

Restriction endonuclease analysis of purified EHV‐1 DNA revealed up to 16 circulating genotypes (Allen, Yeargan, Turtinen, Bryans, & McCollum, 1983). Restriction patterns were generally highly conserved among strains, the main differences being concentrated in the inverted repeat (TRs/IRs) (Binns, McCann, Zhang, Wood, & Mumford, 1994; Studdert, Crabb, & Ficorilli, 1992). Differences in restriction endonuclease patterns within individual isolates have also been observed after passage in cell culture (Allen, Yeargan, & Bryans, 1983; Bonass, Hudson, Elton, Killington, & Halliburton, 1994). Thus, after passage in bovine kidney cells, the Japanese EHV‐1 strain HH1 had mutations in ORF1, ORF24 and ORF71, and the virus lost virulence *in vivo* (Kirisawa, Ui, Takahashi, Kawakami, & Iwai, 1994).

More recent genetic studies include determination of the whole genome sequences for two well‐characterized strains, Ab4 and V592 (Telford et al., 1992; Nugent et al., 2006). Comparison of the sequence of the neuropathogenic strain Ab4 (Crowhurst, Dickinson, & Burrows, 1981; Telford et al., 1992) with that of the abortigenic strain V592 (Mumford et al., 1987; Nugent et al., 2006) identified 43 amino acid residue differences distributed among 31 ORFs (Nugent et al., 2006). Of these, ORF68, which encodes a non‐essential, membrane‐associated virion component homologous to herpes simplex virus type 1 (HSV‐1; species *Human alphaherpesvirus 1*) gene US2, was shown to be particularly variable and was developed as a target for classifying field isolates into six groups (Meindl & Osterrieder, 1999; Nugent et al., 2006). There was no obvious association of these genotypes with pathogenicity.

A single nucleotide polymorphism (A2254G) in ORF30, which encodes the DNA polymerase catalytic subunit, results in the single amino acid substitution N752D and was shown to be associated at a statistically significant level with neurological disease (Nugent et al., 2006). Numerous studies have demonstrated that differences in pathogenicity correlate with the ability to disseminate to and infect vascular endothelial cells in the uterus and central nervous system (Edington, Bridges, & Patel, 1986; Mumford et al., 1994; Patel, Edington, & Mumford, 1982; Platt, Singh, & Whitwell, 1980). Viruses carrying the A2254G neurological marker are thought to replicate to a higher level and induce a longer‐lasting viraemia than those lacking it (Allen & Breathnach, 2006). Welsh mountain ponies infected with a mutant virus expressing N752 induced significantly less neurological disease than the wild‐type virus, which has the D752 neurological marker (Goodman et al., 2007). The presence of the A2254G marker has been linked to increasing morbidity and mortality in field outbreaks since 2000 (Lunn et al., 2009; Smith et al., 2010).

The EHV‐1 pathogenicity determinants that influence abortion are unclear. However, the EHV‐1 neuropathogenic strain Ab4 is significantly better at inducing abortion in experimental studies than other strains, including the abortigenic strain V592 and the neuropathogenic strain OH03 (Mumford et al., 1994; Gardiner et al., 2012). This difference in induced abortion was observed despite the viraemia in infected animals being of similar magnitude, as measured by qPCR (Gardiner et al., 2012). EHV‐1 strains that reproducibly cause neurological disease may also cause abortion, due to high levels of endometrial damage during infection (Gardiner et al., 2012; Smith et al., 1992).

Until recently, only two complete genome sequences have been available for analysis of EHV‐1. The objective of this study was to generate a database of sequence information for a large panel of well‐documented, historically extensive EHV‐1 clinical isolates maintained at the Animal Health Trust (UK), including viruses from respiratory, abortigenic and neurological outbreaks, with the aim of determining whether there are any clear genetic factors involved in the three types of disease. These data would be useful for improving the safety of newly designed modified live vaccines for preventing EHV‐1 disease. The sequences of 78 isolates were determined, covering at least 80% of the genome for each. The majority of strains analysed were low passage clinical isolates from outbreaks in the UK. Together with 26 EHV‐1 genome sequences sequenced in the USA and Australia (Vaz et al., 2016) and deposited in GenBank during the course of this project, these data provide an invaluable database of genetic information for this important pathogen.

## 2 MATERIALS AND METHODS

### 2.1 Viral isolation

EHV‐1 strains (Table 1) were isolated between 1982 and 2016 by the diagnostic laboratories at the Animal Health Trust (UK). For isolation, rabbit kidney (RK13) cells (ATCC^®^ CCL‐37™) were grown in Dulbecco's modified Eagle's medium (DMEM) supplemented with 5% (v/v) heat‐inactivated foetal calf serum, 0.5 mg/ml amphotericin B, 100 IU/ml penicillin and 100 mg/ml streptomycin (PAA Laboratories), and incubated in the presence of 5% (v/v) CO_2_. Viral stocks were clarified by centrifugation and stored at −70°C.

**Table 1:** EHV‐1 strains sequenced

Strain ID (location/isolate/date)	Accession No.	Virus Source	Disease Type	Reads aligned to reference
UK/32/1982	KU206465	Thoroughbred abortion, liver	Abortion	843,272
HONG KONG/57/1984	KU206467	Thoroughbred nasal swab	Respiratory	686,922
SUFFOLK/73/1985	KU206459	Thoroughbred abortion tissue	Abortion	716,506
UK/106/1985	KU206415	Eye swab	Conjunctivitis	615,725
SHROPSHIRE/68/1989	KU206408	Thoroughbred abortion tissue	Abortion	359,673
STAFFORDHIRE/80/1989	KU206436	Thoroughbred abortion, thymus	Abortion	136,510
YORKSHIRE/12/1990	KU206437	Thoroughbred abortion tissue	Abortion	874,231
KENT/177/1991	KU206435	Thoroughbred abortion tissue	Abortion	466,910
YORKSHIRE/1/1993	KU206418	Thoroughbred abortion tissue	Abortion	244,121
BRISTOL/2/1993	KU206451	Abortion, liver	Abortion	319,074
BUCKINGHAMSHIRE/9/1993	KU206428	Thoroughbred abortion, thymus/lung/liver	Abortion	327,543
KENT/43/1994	KU206453	Thoroughbred abortion, thymus/lung/liver	Abortion	688,954
ESSEX/81/1994	KU206472	Thoroughbred abortion, thymus/lung/liver	Abortion	136,139
SUFFOLK/82/1994	KU206409	Thoroughbred nasal swab	Respiratory	299,389
SUFFOLK/91/1994	KU206479	Thoroughbred abortion, lung/liver	Abortion	309,486
UK/109/1994	KU206434	Thoroughbred abortion, thymus/lung/liver	Abortion	389,104
SUFFOLK/110/1994	KU206460	Thoroughbred abortion, thymus/lung/liver	Abortion	145,259
CAMBRIDGESHIRE/3/1995	KU206432	Thoroughbred abortion tissue	Abortion	200,405
UK/58/1995	KU206414	Thoroughbred abortion tissue	Abortion	373,273
BERKSHIRE/7/1996	KU206463	Thoroughbred abortion tissue	Abortion	222,162
LINCOLNSHIRE/10/1996	KU206412	Thoroughbred abortion tissue	Abortion	745,418
LINCOLNSHIRE/13/1996	KU206416	Thoroughbred abortion tissue	Abortion	385,048
SUFFOLK/16/1996	KU206417	Thoroughbred abortion tissue	Abortion	336,392
LEICESTERSHIRE/22/1996	KU206464	Nasal turbinate tissue	Paralysis	385,089
SHORPSHIRE/38/1996	KU206407	Thoroughbred abortion tissue	Abortion	349,642
WILTSHIRE/40/1996	KU206421	Thoroughbred abortion tissue	Abortion	1,046,193
LEICESTERSHIRE/59/1996	KU206423	Thoroughbred abortion tissue	Abortion	326,111
SUFFOLK/60/1996	KU206422	Thoroughbred abortion tissue	Abortion	300,516
GLOUCESTERSHIRE/127/1998	KU206445	Heparinized blood	Ataxia	736,301
YORKSHIRE/114/1999	KU206473	Heparinized blood	Abortion (in contact)	968,797
LEICESTERSHIRE/13/2000	KU206413	Abortion, tissue	Abortion	854,123
DERBYSHIRE/39/2002	KU206429	Abortion, tissue	Abortion	474,733
DEVON/28/2003	KU206440	Donkey, heparinized blood	Respiratory (in contact)	1,649,625
UK/58/2003	KU206444	Thoroughbred, heparinized blood	Ataxia (in contact)	158,798
NOTTINGHAMSHIRE/10/2004	KU206404	Abortion, tissue	Abortion	981,356
BRISTOL/55/2004	KU206424	Abortion, tissue	Abortion	742,045
NOTTINGHAMSHIRE/70/2004	KU206420	Abortion, tissue	Abortion	160,936
SUFFOLK/48/2005	KU206430	Nasopharyngeal swab	Respiratory	509,536
SUFFOLK/123/2005	KU206480	Cob abortion, lung/liver/thymus/spleen	Abortion	412,587
ESSEX/199/2005	KU206410	Thoroughbred abortion tissue, lung/liver/thymus/spleen	Abortion	560,623
ESSEX/200/2005	KU206411	Abortion, tissue	Abortion	1,103,748
SHROPSHIRE/167/2006	KU206405	Foal tissue	Vaccinated mare stillbirth	574,048
HAMPSHIRE/1/2008	KU206462	Thoroughbred abortion tissue, lung/liver/thymus/spleen	Abortion	925,610
BERKSHIRE/64/2009	KU206438	Abortion, tissue	Abortion	281,139
HAMPSHIRE/77/2009	KU206419	Abortion, tissue	Abortion	1,149,655
SUFFOLK/87/2009	KU206443	Abortion, tissue	Abortion	629,670
HERTFORDSHIRE/109/2009	KU206476	Thoroughbred Foal tissue	Died one day post partum	642,031
BUCKINGHAMSHIRE/114/2010	KU206433	Abortion, tissue	Abortion	1,036,214
NORFOLK/124/2010	KU206427	Thoroughbred abortion tissue	Abortion	316,420
HERTFORDSHIRE/188/2010	KU206466	Thoroughbred Cross, Nasopharyngeal swab	Ataxia	314,104
CHESHIRE/275/2010	KU206406	Thoroughbred abortion, placenta	Abortion	1,062,479
OXFORDSHIRE/27/2011	KU206454	Thoroughbred spinal cord	Ataxia	830,283
OXFORDSHIRE/31/2011	KU206456	Thoroughbred abortion foetus tissue	Abortion	537,157
OXFORDSHIRE/34/2011	KU206457	Thoroughbred abortion, placenta	Abortion	1,235,589
BUCKINGHAMSHIRE/93/2011	KU206455	Thoroughbred abortion tissue, lung/liver/thymus/spleen	Abortion	532,780
LINCOLNSHIRE/2/2012	KU206475	Thoroughbred abortion tissue	Abortion	764,422
SUFFOLK/10/2012	KU206474	Thoroughbred abortion tissue	Abortion	557,628
BUCKINGHAMSHIRE/24/2012	KU206431	Warmblood abortion tissue	Abortion	608,214
DEVONSHIRE/97/2012	KU206469	Thoroughbred heparinized blood	Pyrexia (ataxia in contact)	349,099
GLOUCESTERSHIRE/54/2013	KU206447	Hunter heparinized blood	Ataxia	828,984
SUFFOLK/41/2013	KU206458	Thoroughbred abortion tissue	Abortion	656,506
SUFFOLK/45/2013	KU206452	Thoroughbred abortion tissue/placenta	Abortion	639,154
SUFFOLK/48/2013	KU206425	Thoroughbred abortion tissue/placenta	Abortion	715,818
GLOUCESTERSHIRE/70/2013	KU206448	Warmblood heparinized blood	Ataxia	1,047,393
GLOUCESTERSHIRE/77/2013	KU206446	Warmblood nasopharyngeal Swab	Ataxia	1,086,100
SUFFOLK/82/2013	KU206441	Thoroughbred abortion tissue/placenta	Abortion	823,706
ABERDEENSHIRE/84/2013	KU206461	Abortion, tissue	Abortion	1,086,568
SUFFOLK/89/2013	KU206442	Abortion, tissue	Abortion	875,004
OXFORDSHIRE/206/2013	KU206470	Thoroughbred nasopharyngeal swab	Ataxia, euthanased	1,106,795
OXFORDSHIRE/207/2013	KU206471	Thoroughbred heparinized blood	Ataxia (in contact)	962,635
CAMBRIDGESHIRE/96/2013	KU206439	Thoroughbred abortion tissue	Abortion	964,679
GLOUCESTERSHIRE/108/2013	KU206449	Thoroughbred nasal swab	Ataxia (in contact)	917,254
GLOUCESTERSHIRE/114/2013	KU206450	Thoroughbred heparinized blood	Ataxia	990,750
SUFFOLK/125/2013	KU206426	Thoroughbred abortion tissue	Abortion	435,435
HERTFORDSHIRE/150/2016	KY852346	Thoroughbred abortion tissue	Abortion	1,348,968
ARMY 183 ‐ 1941	KU206477	Historical respiratory isolate	Respiratory	514,803
AB1 ‐ 1979	KU206468	Thoroughbred abortion tissue	Abortion	114,868
RACL11 ‐ 1950s	KU206478	Abortion tissue	Abortion	40,204

### 2.2 Preparation of viral DNA

The virus stocks were titrated by plaque assay on RK13 cells as described previously (Tearle et al., 2003). Viral DNA was extracted from semi‐purified nucleocapsids as follows. RK13 cells were infected at a multiplicity of infection of 0.1 plaque‐forming units per cell and incubated at 37°C in 5% (v/v) CO_2_ until 80% of cells showed cytopathic effect (4–6 days). Cells were harvested and viral nucleocapsids were enriched using a variation of a protocol described previously (Szpara, Tafuri, & Enquist, 2011). Cell pellets from two 150 cm^2^ flasks were resuspended in 5 ml LCM buffer (3% sodium deoxycholate, 30 mM Tris‐HCl pH 7.4, 123 mM KCl, 0.5 mM EDTA, 3.6 mM CaCl_2_, 3 nM MgCl_2_, 3% (v/v) NP‐40 and 43 μl 2‐mercaptoethanol/100 ml), and lipid envelopes were removed by adding 1 ml 1,1,2‐trichloro‐1,2,2‐trifluroethane (Freon, Sigma‐Aldrich) and mixing by inversion. The phases were separated by centrifugation at 1,500 *g* for 5 min, and the supernatant was re‐extracted with 1 ml Freon. Approximately 2.8 ml supernatant was layered over a two‐step glycerol gradient consisting of 1.3 ml 45% (v/v) and 1.3 ml 5% (v/v) glycerol in LCM buffer in a 5‐ml thin‐walled Ultraclear centrifuge tube (Beckman Coulter). Viral nucleocapsids were pelleted by centrifugation at 96,000 *g* in an AH‐650 rotor for 1 hr at 4°C. The glycerol was removed, and the pellet was drained by inverting the tube and resuspended in 400 μl sterile phosphate‐buffered saline. DNA was extracted from the enriched viral nucleocapsids using an Isolate II genomic DNA kit (Bioline) according to the manufacturer's instructions, and eluted in 100 μl water. DNA concentration was quantified using a Nanodrop (Thermo Scientific), and DNA integrity was assessed by agarose gel electrophoresis of intact DNA.

### 2.3 Preparation of DNA sequencing libraries

Libraries for EHV‐1 strains to be sequenced at the Animal Health Trust (UK) were prepared using an Illumina series KAPA library preparation kit (catalogue no. KK8200). An aliquot (200 ng) of viral DNA was sheared by sonication and used as input for the library preparation protocol as described in the manufacturer's instructions. A MinElute reaction clean‐up kit (Qiagen) was used to purify the DNA between steps, and DNA fragments of approximately 500 bp were selected by agarose gel electrophoresis. DNA bands were excised and purified using a QIAquick gel extraction kit (Qiagen).

The Genome Analysis Centre (now the Earlham Institute; UK) prepared viral DNA libraries using a Covaris S2 sonicator and an Illumina TruSeq DNA library preparation kit as described by the manufacturer. Size selection of 500‐bp DNA fragments was performed using E‐Gel^®^ SizeSelect™ 2% (w/v) agarose gels (Invitrogen). The insert size of the libraries was verified using an Aligent 2100 bioanalyser and a DNA 1000 assay (catalogue no. 5067‐1504). DNA concentration was determined using a Qubit^®^ dsDNA HS assay kit (catalogue no. Q32854).

### 2.4 DNA sequencing

Paired‐end read sequencing runs of 75–250 nt/read were carried out on an Illumina MiSeq sequencer, using the MiSeq reagent kit v. 2 or v. 3 (300–500 cycles). Following preliminary analysis, the MiSeq reporter programme was used to generate FASTQ‐formatted read files for each EHV‐1 strain.

### 2.5 Assembly of DNA sequence data

Read data were assessed for quality using FastQC (http://www.bioinformatics.babraham.ac.uk/projects/fastqc/) running within Bio‐Linux 7 (Field et al., 2006). Adapter sequences were removed from the reads, which were then quality filtered and trimmed using Trim Galore v. 0.3.3 (http://www.bioinformatics.babraham.ac.uk/projects/trim_galore/). If necessary, the 3′‐ends of the reads were then trimmed using FASTQ/A trimmer, which is part of the FASTX‐toolkit (http://hannonlab.cshl.edu/fastx_toolkit/).

For *de novo* assembly, host cell DNA reads were removed using BWA v. 0.5.9rc1 (Li & Durbin, 2010) in paired‐end mode to align to the rabbit genome (Ensembl: Oryctolagus_cuniculus.OryCun2.0.73.dna_sm.toplevel.fa). Unmapped reads were extracted from the SAM file using bam2fastq (http://gsl.hudsonalpha.org/information/software/bam2fastq) and assembled into contigs using ABYSS v. 1.3.7 (Simpson et al., 2009). The contigs were oriented using ABACAS (Assefa, Keane, Otto, Newbold, & Berriman, 2009) with EHV‐1 strain V592 (GenBank: AY464052) as the reference genome. The reads were aligned to the resulting scaffold using BWA v. 0.5.9rc1. SAM file alignments were visualized in Unipro Ugene v. 1.13.1 (Okonechnikov, Golosova, & Fursov, 2012) or Tablet v. 1.13.12.17 (Milne et al., 2013), and errors were corrected manually.

Reference‐guided assemblies were conducted on quality‐trimmed reads using BWA v. 0.5.9rc1, using strain V592 or Ab4 as the reference. Consensus sequences were determined in Ugene (Okonechnikov et al., 2012) and exported as FASTA files. The final DNA sequences were annotated using GATU (Tcherepanov, Ehlers, & Upton, 2006). The EHV‐1 strain V592 (accession number AY464052) annotation was used to create a feature table that was then propagated to all sequences in a FASTA format sequence alignment created by MAFFT v. 7.157b (Katoh & Standley, 2013). For deposition of sequences in GenBank, sequence files were assembled with strings of 100 N residues between blocks of contiguous data. Equivalent blocks of EHV‐1 sequences were trimmed to the same length for the analysed strains.

### 2.6 Phylogenetic and recombination analyses

Nucleotide sequences were aligned using MAFFT v. 7.157b (Katoh & Standley, 2013), and the alignments were visualized using Jalview v. 2.8.1 (Waterhouse, Procter, Martin, Clamp, & Barton, 2009). Gaps, low coverage regions and tandem repeats were removed, so that all sequences could be compared. The sequences of individual ORFs were extracted and then translated using EMBOSS Transeq (http://www.ebi.ac.uk/Tools/st/emboss_transeq/), and the resulting amino acid sequences were aligned using MAFFT v. 7.157b (Katoh & Standley, 2013). Maximum likelihood (ML) phylogenetic trees were constructed using PhyML v. 3.1 (Guindon et al., 2010) under the general time reversible substitution model, as determined by jModeltest (Posada, 2009), with branch swapping by tree‐bisection‐reconnection. One hundred bootstrap replicates were conducted to assess statistical support for the tree topology. Phylogenetic networks were constructed using Splitstree v. 4 (Huson & Bryant, 2006), using the uncorrected P characters transformation. Regions of potential recombination with EHV‐4 and EHV‐8 were also investigated by identifying individual sequence reads having low sequence conservation to the reference sequence and using them to query the non‐redundant nucleotide database in GenBank, employing the Basic Local Alignment Search Tool (BLAST) (Altschul, Gish, Miller, Myers, & Lipman, 1990) (http://blast.ncbi.nlm.nih.gov/Blast.cgi). Sequences were obtained from GenBank and used in Simplot v. 3.5.1 to visualize regions of recombination (Lole et al., 1999). RDP v. 4 was used to investigate EHV‐1 recombination further, using default settings (Martin et al., 2010).

The sequences of the following EHV‐1 strains or isolates (with GenBank accession numbers) derived by others were included in the analysis: HH1 (AB992258), 1074‐94 (KT324730), NY03 (KF644569), 717A‐82 (KT324733), 3045‐07 (KT324725), 00c19 (KF644576), 89c105 (KF644577), 89c25 (KF644579), 90c16 (KF644566), 01c1 (KF644578), T953 (Findlay) (KM593996), OH03 (KF644571), 1029‐93 (KT324731), NY05 (KF644570), 2222‐03 (KT324727), NZA‐77 (KT324724), VA02 (KF644572), FL06 (KF644567), 970‐90 (KT324732), 3038‐07 (KT324726), 1966‐02 (KT324729), 2019‐02 (KT324728), 438‐77 (KT324734), NMKT04 (KF644568), Ab4 (AY665713) and V592 (AY464052).

Protein structures were annotated using UCSF Chimera version 1.11 (Pettersen et al., 2004) and the HSV‐1 DNA polymerase catalytic subunit (Protein Data Bank number 2gv9) (Liu et al., 2006), using amino acid numbering obtained from pairwise alignments of the EHV‐1 strain V592 and HSV‐1 DNA polymerase catalytic subunits (accession numbers Q6S6P1 and AB691552, respectively).

## 3 RESULTS

### 3.1 DNA sequencing of EHV‐1 strains

Seventy‐eight representative EHV‐1 isolates selected from the archive at the Animal Health Trust (UK) were sequenced (Table 1). A sequencing library was prepared for each strain using one of two approaches, as described in materials and methods, generating paired‐end reads (i.e., sequences from the opposing ends of random DNA fragments) of 75–250 nucleotides (nt) each.

Methods involving *de novo* and reference‐guided assembly were then used to create contigs covering the majority of U_L_ and U_S_ as indicated in Figure 1. The use of *de novo* assembly indicated that the genome structure was conserved in the viruses sequenced, in that no large‐scale rearrangements were identified. The number of reads mapped to the reference genome for each virus is shown in Table 1.

As for other herpesvirus genomes, several regions of tandem repeats were identified (Figure 1). The tandem repeat regions were difficult to sequence as they were often longer than the read length. Even when tandem repeats were short enough to be covered within a single read, they were observed to vary in length among strains, and sometimes within strains, as reported previously for EHV‐1 (Nugent et al., 2006) and other alphaherpesviruses (Depledge et al., 2014; Hondo & Yogo, 1988). They also tended to be G+C‐rich and thus seemed to be under‐represented in the sequence libraries, making it inadvisable to estimate their lengths from coverage density data. To estimate the lengths of tandem repeats, the sizes of PCR products were measured by gel electrophoresis. However, attempts to define precise lengths by sequencing these products often failed. Therefore, sequence files were constructed as gapped sequences, with strings of 100 N residues replacing the tandem repeats to signify their undetermined length and sequence.

### 3.2 Phylogenetic analysis of EHV‐1

**Figure 2:**
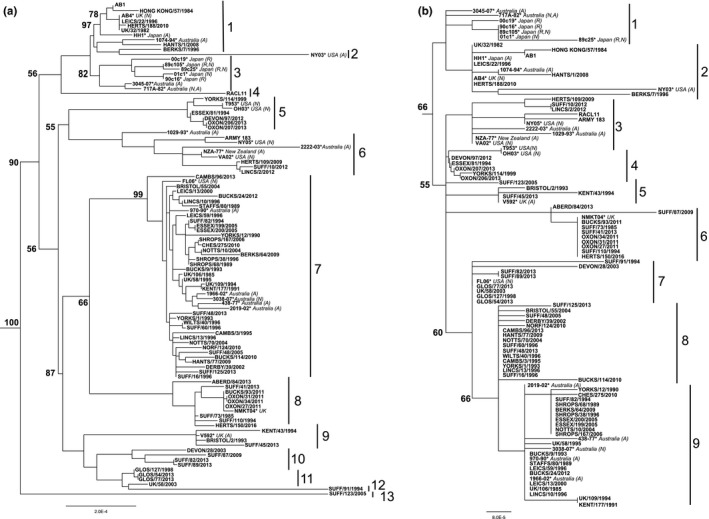
ML phylogenetic trees of (a) EHV‐1 U_L_ and (b) EHV‐1 U_S_, midpoint rooted. Bootstrap values obtained after 100 replicates are shown at the major nodes. Clades are indicated by continuous bars on the right and numbered. Accession numbers and disease phenotype for new sequences are listed in Table 1. Sequences obtained from PubMed are indicated with an asterisk (*), country of isolation is noted in italics, and disease phenotypes are indicated in parentheses as respiratory (R), neurological (N) or abortigenic (A) if known

Phylogenetic analysis was conducted on 111,154 bp containing the majority of U_L_ and 11,636 bp from U_S_. This involved data for 75 of the strains (not including the majority of identical strains from individual outbreaks) listed in Table 1, together with 26 sequences obtained from GenBank from viruses isolated in Australia, Japan, UK and USA. Gaps, ambiguous sites, variable regions and tandem repeats were removed before analysis. The U_L_ tree demonstrated the existence of 13 clades supported by high bootstrap values (Figure 2a). Overall sequence conservation was high, strain Ab1 (clade 1) having 99.72% identity to strain Suffolk/123/2005 (clade 13). The majority of the strains isolated in the UK grouped in clade 7, along with five Australian isolates and one from the USA. U_S_ separated into nine clades, with members grouped largely similarly to the clades identified when using U_L_ (Figure 2b). Sequence conservation was even higher in U_S_ than U_L_, with 99.89% identity between strains Ab1 (clade 2) and Suffolk/87/2009 (clade 6).

### 3.3 Genetic variability in EHV‐1

Analysis of the ORFs from the clinical isolates identified mainly single nucleotide substitutions and a few short deletions in both coding and non‐coding regions, leading to truncations of four ORFs in strains isolated from abortions. Strain Kent/43/94 had a nucleotide substitution at gene position 1558 of a cytosine to a thymine, resulting in a nonsense codon and a 12 amino acid *C*‐terminal truncation in ORF63 (encoding ICP0). Strain Buckinghamshire/9/93 had a frameshift in ORF34 (protein V32), caused by a deletion of nucleotides 231‐232, within the dinucleotide repeats ^231‐^AGAGAG^‐236^, which resulted in amino acid residues 79–123 being translated in another reading frame, in comparison with the full‐length protein of 160 residues. Strain Cambridgeshire/96/2013, which was isolated from a pregnant mare imported from northern France, had a single nucleotide deletion of a cytosine at position 1,853 in a short four nucleotide homopolymeric tract in ORF14 (VP11/12), generating a premature stop codon and a truncated protein of 626 residues compared to the full‐length protein of 750 residues. Strain Suffolk/45/2013 had a single nucleotide deletion of a guanine at nucleotide position 574 in a short four nucleotide homopolymeric tract in ORF11 (VP22), leading to a premature stop codon, which truncated the protein from 304 to 205 residues. The strain RacL11 had a deletion between ORF1 and ORF2, resulting in an ORF1 protein of 18 amino acid residues and the total loss of ORF2. This strain had an intact ORF67 as previously reported, unlike the vaccine strain RacH derived from it (Ma, Feineis, Osterrieder, & Van de Walle, 2012; Reczko & Mayr, 1963).

There was no evidence in the sequence data from the viral stocks used for the loss of a BamHI site in ORF21 (ribonucleotide reductase large subunit), as was reported after serial passage of strains Army 183 and Ab1 in cell culture (Bonass et al., 1994). Unfortunately, the passage number of these stocks was unknown.

**Figure 3:**
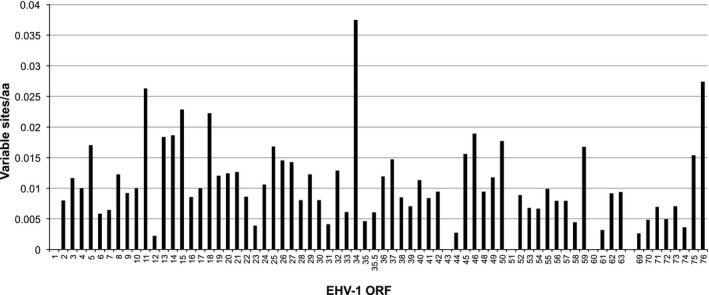
Variation in EHV‐1 protein sequences. The mean number of amino acid (aa) substitutions (relative to the consensus), normalized to protein length, is shown for the 78 strains. The ORF number is indicated on the *x*‐axis. The mean number of substitutions for the combined ORFs is marked by a dotted line. ORF64‐ORF68 were not included, due to low sequence coverage for several strains. Nonsense mutations were counted as single substitutions for this analysis, and frameshifts were not included. Repeat regions are not included because of variation within strains and low sequence coverage

To investigate protein sequence divergence, the numbers of variable amino acids in ORF1 to ORF63 (in U_L_) and ORF69 to ORF76 (in U_S_), normalized to protein length but excluding frameshifts and truncations, were counted in the newly sequenced viruses (Figure 3). The most variable proteins were ORF34 (protein V32), ORF76 (membrane protein US9), ORF11 (VP22), ORF15 (protein UL45) and ORF18 (DNA polymerase processivity subunit). Complete amino acid sequence conservation was observed in ORF1 (protein UL56), ORF43 (capsid triplex subunit 2), ORF51 (myristylated tegument protein) and ORF60 (nuclear protein UL3) in all strains apart from RacL11, which lacks the majority of ORF1 and ORF2.

**Figure 4:**
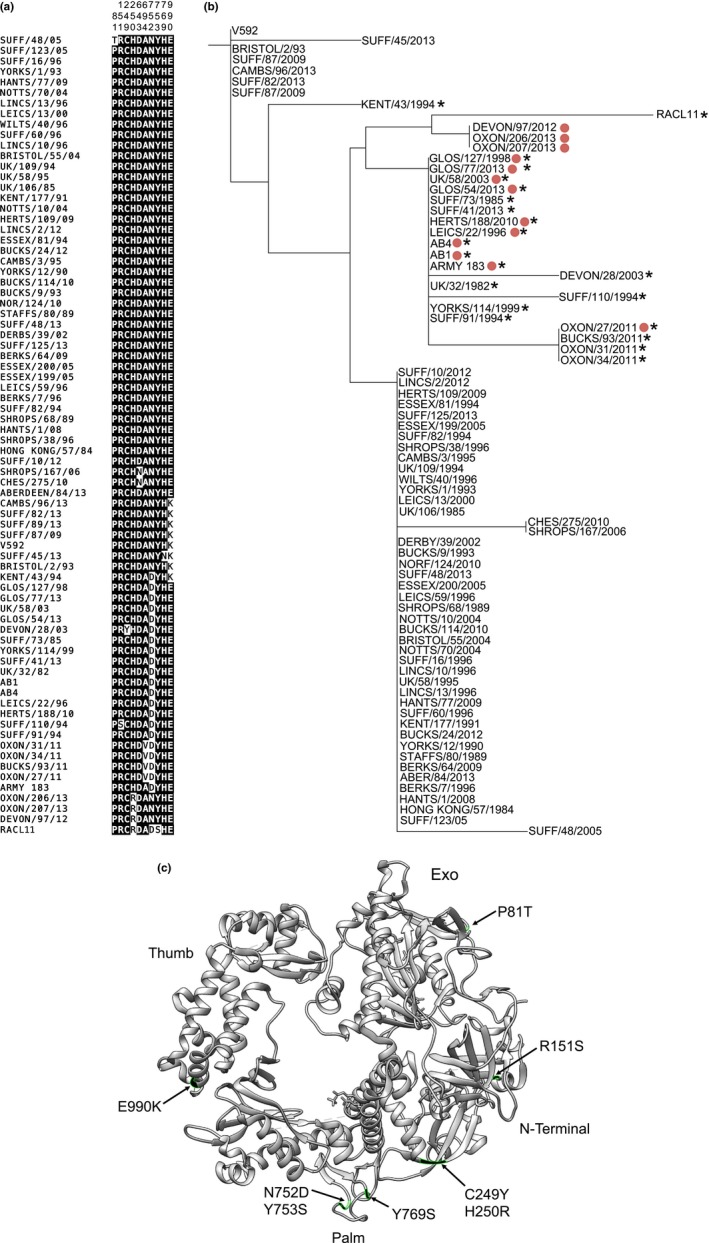
Mutations in the DNA polymerase catalytic subunit (ORF30). (a) Variable amino acid residues for EHV‐1 strains are shown in a sequence alignment and are identified by residue numbering at the top. (b) ML tree of the amino acid sequences created using PhyML v 3.1. Strains isolated from neurological disease outbreaks are identified by red circles, and strains with the G2254/D752 genotype are identified by asterisks. (c) Ribbon diagram of the crystal structure of HSV‐1 DNA polymerase catalytic subunit (2gv9.pdb) with mutations mapped on the surface. P81T in EHV‐1 corresponds to position 102 in HSV‐1, R151S to position 175, C249Y to position 254 and H250R to position 255. Residues 643 and 694 are not resolved and are therefore omitted. N752D corresponds to position 751, Y753S to position 752, Y769 to position 768 and E990K to position 989 [Colour figure can be viewed at http://wileyonlinelibrary.com]

In this study, a total of 10 variable sites were identified in the DNA polymerase catalytic subunit (total length 1,220 amino acid residues) (Figure 4a). The majority of viruses associated with neurological disease outbreaks had the G2254/D752 genotype, with the notable exception of strain Devon/97/2012 and the related viruses represented by Oxford/206/2013, as shown in the ML tree (Figure 4b). These viruses also had the substitution H250R within the protein. Interestingly, the low virulence strain V592 and a cluster of genetically related strains from clades 6, 8 and 9 in the U_L_ tree (Figure 2a) had an alternative substitution at E990K, towards the *C*‐terminus. Mapping of the 10 polymorphisms indicated in Figure 4c onto the three‐dimensional structure of the HSV‐1 DNA polymerase catalytic subunit (2gv9.pdb) showed that the variant residues were located in the palm, *N*‐terminal, exo and thumb domains.

### 3.4 Recombination in EHV‐1

**Figure 5:**
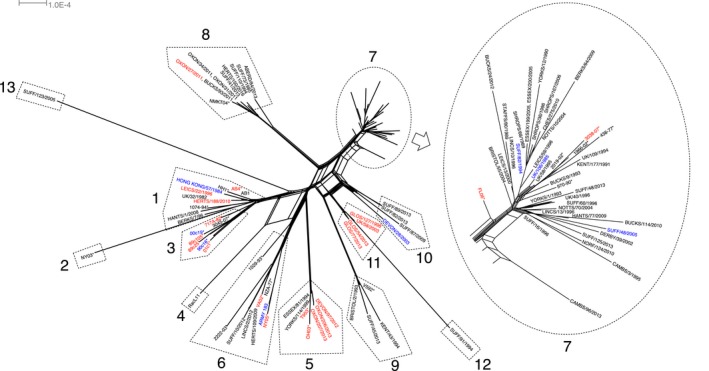
Phylogenetic network based on EHV‐1 U_L_, created using Splitstree v. 4. Clades are indicated as in Figure 2a. Clade 7 includes a magnified view. Sequences obtained from PubMed are labelled with an asterisk (*). Viruses isolated from respiratory disease or conjunctivitis outbreaks are coloured blue, neurological disease outbreak viruses are coloured red, and viruses from abortions are coloured black [Colour figure can be viewed at http://wileyonlinelibrary.com]

The Splitstree program was used to assess whether there was evidence for recombination in EHV‐1, as a tree‐like splits network can be interpreted as supporting various modes of evolution, including recombination (Huson, 1998; Huson & Bryant, 2006). A splits network was constructed using sequence data from U_L_ for all strains, employing the default NeighborNet method implemented in Splitstree v. 4 (Figure 5). The network showed the existence of 13 clades, arranged very similarly to the clades identified in the ML phylogenetic analysis (Figure 2a). The four outlier strains (Suffolk/91/94, Suffolk/123/05, NY03 and RacL11) may represent single representatives of EHV‐1 clades and have been numbered accordingly. Statistically, the splits network gave a least squares fit of 98.587%, suggesting that the data are relatively tree‐like, which may suggest that recombination has played a role in the evolution of EHV‐1. A splits network constructed using the split decomposition method produced essentially identical results (data not shown). As before, the majority of UK isolates fell within one clade.

**Figure 6:**
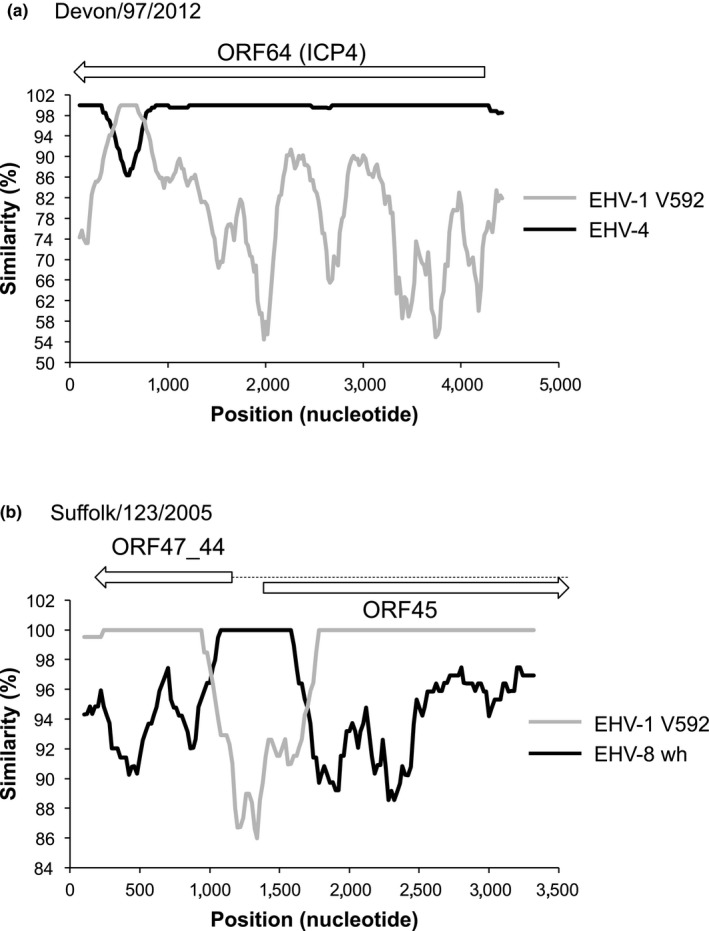
Recombination in EHV‐1. Similarity plots of selected regions of EHV‐1 strains (a) Devon/97/2012 (nucleotides 113835‐118298) compared to EHV‐4 strain NS80567, (b) Suffolk/123/2005 (nucleotides 83081‐86533) compared to EHV‐8 strain wh, analysed by Simplot. Arrows indicate the position and orientation of ORFs in relation to the regions of recombination, with the spliced ORF47_44 indicated with a dotted line

Recombination events potentially involving other equine alphaherpesviruses were identified initially by viewing BWA alignments in Tablet (Milne et al., 2010), focusing on regions in which the sequence reads aligned poorly with the EHV‐1 references. Subsequent analysis of these regions using the program Simplot v.3.5.1 (Lole et al., 1999) produced sequence similarity plots that highlighted regions of possible recombination. Strain Devon/97/2012 contained a region at 114,261–114,609 bp (strain V592 numbering), within ORF64 (ICP4, GenBank accession number MG256761), that was closely related to the corresponding region in EHV‐4 strain NS80567 (GenBank accession AF030027) (Figure 6a). This sequence was also present in strain Oxford/206/2013, which was linked anecdotally to the earlier Devon virus outbreak. Analysis of sequence data from GenBank identified this sequence with 100% identity in EHV‐1 strains T953 (Findlay) (GenBank accession KM593996) and OH03 (GenBank accession KF644571). Strain Suffolk/123/2005 had multiple substitutions at 84,158–84,778 bp (strain V592 numbering), encompassing the splice site at the beginning of ORF44 (ORF47/44 encodes DNA packaging terminase subunit 1) and the start codon of ORF45 (DNA packaging tegument protein UL17). This region was identical to the corresponding region in EHV‐8 strain wh (GenBank accession JQ343919) (Figure 6b). This recombination event was also detected using the program RDP4 (Martin et al., 2010) (data not shown). The amino acid sequence of ORF47/44 was conserved with the other EHV‐1 strains. However, 3 amino acid substitutions were present in the *N*‐terminal region of the ORF45 protein as a result of this apparent recombination event.

## 4 DISCUSSION

The apparent association of G2254/D752 strains, rather than A2254/N752 strains, with neuropathogenic disease has been a particular focus of studies of EHV‐1 pathogenesis in recent years (Fritsche & Borchers, 2011; Pronost et al., 2010; Stasiak, Rola, Ploszay, Socha, & Zmudzinski, 2015; Tsujimura et al., 2011). Sometimes overlooked is the finding in the field that the association of abortion with the EHV‐1 A2254/N752 strain variant was more statistically significant (Nugent et al., 2006). The majority of strains analysed in the present study had been isolated from abortion cases and had the A2254/N752 genotype, and most of the remaining strains that had been isolated from neurological disease outbreaks belonged to the G2254/D752 genotype, similar to published conclusions. However, there are other genetic differences between these genotypes within the DNA polymerase catalytic subunit, and their effects on protein function have yet to be studied.

The previously sequenced EHV‐1 strains Ab4 (G2254; clade 1) and V592 (A2254; clade 9) can both cause abortion in an experimental setting, albeit with apparently different efficiencies. Infection of pregnant ponies and horses with strain Ab4 resulted in severe clinical disease, with high levels of viral shedding and pyrexia in all ponies, coupled with abortion, ataxia and quadriplegia (Gardiner et al., 2012; Mumford et al., 1994). Experimental infections with strain V592 induced mild respiratory signs and low numbers of abortions, but not neurological disease (Mumford et al., 1994). The duration of viraemia and the amount of viral shedding was also significantly lower in horses infected with V592 compared with Ab4 (Mumford et al., 1994; Smith et al., 2000). Despite the virulence of D752 strains, the majority of disease in equines does not appear to be caused by them, with 73% of the viruses sequenced in the present study not having the marker.

Previously published work has shown no obvious difference in the processivity of either DNA polymerase variant *in vitro*, except that the G2254 version was sensitive to the drug aphidicolin (Goodman et al., 2007). This may indicate that the two versions of the protein adopt slightly different conformations that affect pathogenicity by a means that has not yet been identified. It has been suggested that G2254 strains are able to infect cell types *in vivo* that are different from those infected by A2254 strains, allowing these viruses to replicate and disseminate more efficiently (Goodman et al., 2007). However, work carried out *in vitro* has failed to identify any cellular preference due to this mutation (Ma, Lu, & Osterrieder, 2010).

Interestingly, we found a polymorphism in ORF30 in a subset of viral strains responsible for neurological outbreaks during 2012–2013. Strain Devon/97/2012 (A2254) caused neurological disease in multiple Thoroughbred horses, resulting in euthanasia in some cases. Epidemiological analysis suggested that this outbreak was linked to a later one in Oxfordshire, which again caused severe neurological disease. Analysis of ORF30 identified the single substitution H250R (Figure 4) located in the *N‐*terminal domain of the DNA polymerase catalytic subunit adjacent to a cluster of conserved, highly charged residues that may interact with RNA (Liu et al., 2006). This substitution was also found in the strain RacL11, along with two further changes in ORF30, the neurological marker N752D and Y753S. The polymorphism at position 753 has been reported previously in a subset of strains isolated in central Kentucky (Smith et al., 2010) and in the strain RacH used in the live attenuated vaccines Rhinomune (Boehringer Ingelheim Vetmedica) and Prevaccinol (MSD Animal Health) (Nugent et al., 2006).

During latency, alphaherpesvirus genomes exist in a circular, episomal form in the cell nucleus. There is no viral replication and viral transmission does not occur. This process is likely to slow down the evolutionary rate of the virus in individual animals. Moreover, recent work has suggested that host cell‐encoded DNA repair mechanisms are able to control genetic damage in latent herpesvirus genomes (Brown, 2014). Sequencing of live attenuated varicella‐zoster virus (VZV) vaccine isolates from individuals has further implied that latency slows the evolutionary rate (Weinert et al., 2015). The phylogenetic tree for EHV‐1 U_L_ (Figure 2a) indicates that the various clades have not evolved linearly over the last 35 years, but rather that simultaneous co‐circulation of all the clades has occurred. For example, strain Gloucestershire/127/1998 is very similar to two recently isolated strains, Gloucestershire/77/2013 and Gloucestershire/54/2013 which all cluster in clade 11 (Figure 2a). All three samples were obtained from the same geographical area and submitted for diagnosis by the same veterinary practice, suggesting that this strain may have been maintained in the horse population at that area for at least 15 years.

The dynamics of EHV‐1 latency and reactivation are not well understood. Latent virus has been identified in lymph nodes and nervous tissue (including trigeminal ganglia), and latency‐associated transcripts have been identified in CD5+/CD8+ leucocytes (Baxi et al., 1995; Chesters, Allsop, Purewal, & Edington, 1997; Pusterla, Mapes, & Wilson, 2010). The amount of subclinical reactivation from these sites and the mechanisms of subsequent clearance are unknown. The anatomically distinct nature of these sites of latency raises the possibility that viruses at different sites may reactivate independently, and that the overall population may have been derived from viruses originating from separate shedding episodes, similar to that reported for herpes simplex virus 2 (HSV‐2) (Johnston et al., 2014). In the present study, strains Essex/199/05 and Essex/200/05 were isolated from different tissues from an individual abortion and had identical sequences, as expected (Figure 2a, clade 7). Strains Nottinghamshire/10/04 and Nottinghamshire/70/04 were also isolated from pooled tissue from one abortion, but were genetically different from each other (Figure 2a, clade 7), suggesting that horses can be infected with multiple EHV‐1 strains at the same time. This could create difficulties when assigning a particular disease presentation to a viral strain, as some strains may be coincidental isolations.

Co‐infection also gives rise to the possibility of recombination to generate new strains. High‐throughput genome sequencing has provided evidence of recombination between strains in other herpesviruses, namely HSV‐2, VZV, Marek's disease virus, HSV‐1, pseudorabies virus, human cytomegalovirus and infectious laryngotracheitis virus (Hughes & Rivailler, 2007; Kolb, Larsen, Cuellar, & Brandt, 2015; Lee et al., 2013; Norberg et al., 2011, 2015; Sijmons et al., 2015; Szpara et al., 2014; Szpara, Tafuri, et al., 2011; Zell et al., 2012). In the present study, network analysis suggested that recombination has also occurred between EHV‐1 strains. In addition, recombination between EHV‐1 and other, closely related equine herpesviruses was also detected, indicating that co‐infection of the same cells with the parental viruses had occurred. Strain Devon/97/2012 and the two epidemiologically linked strains Gloucestershire/77/2013 and Gloucestershire/54/2013 shared a sequence in ORF64 (ICP4) encoding 116 amino acid residues (1231‐1347) (Grundy, Baumann, & O'Callaghan, 1989) that is more closely related to the corresponding region of EHV‐4 strain NS80567 than other EHV‐1 strains. ICP4, the sole immediate–early protein in EHV‐1, is essential for viral replication (Garko‐Buczynski, Smith, Kim, & O'Callaghan, 1998). There were four non‐synonymous changes in this region when compared to the consensus EHV‐1 sequence, but it is not known whether this has any functional outcome, although the *C*‐terminus of HSV‐1 ICP4 is thought to enhance the functions of the transactivation domain located at the *N*‐terminus (Bruce & Wilcox, 2002; Wagner, Bayer, & Deluca, 2013). A similar, but not identical, recombination event between EHV‐1 and EHV‐4 has been reported in the same ORF in an EHV‐1 strain isolated in Japan (Pagamjav, Sakata, Matsumura, Yamaguchi, & Fukushi, 2005). In addition, strain Suffolk/123/2005, isolated from an abortion, had a sequence of 620 bp containing the ORF47/44 splice acceptor site and the 5′‐end of ORF45 that is identical to the corresponding region in EHV‐8 (Figure 6). EHV‐8 was first isolated from donkeys treated with corticosteroids, probably as a result of viral reactivation from latency, and was initially named asinine herpesvirus 3 (Browning, Ficorilli, & Studdert, 1988). EHV‐8 strain wh, for which a complete genome sequence is available, was isolated from a horse in China in 2010 (Liu, Guo, Lu, Xiang, & Wang, 2012), suggesting that EHV‐8 is not specific to its host species. One EHV‐1 strain sequenced in this report, Devon/28/2003, which falls within clade 10 in the ML tree (Figure 2a), was isolated from a donkey with respiratory disease. It has been reported previously that it is difficult to infect donkeys experimentally with EHV‐1 derived from a horse (Gupta et al., 2000). However, recent reports from Ethiopia of severe disease in donkeys caused by EHV‐1 suggest that these animals can be productively infected naturally and may be able to shed virus in a similar way to that reported for mules, thus posing a threat to other equines in close proximity (Negussie, Gizaw, Tessema, & Nauwynck, 2015; Pusterla et al., 2012).

The viruses sequenced in this report were isolated in cell culture over a period of 35 years. Passage of EHV‐1 (Allen, Yeargan, et al., 1983; Bonass et al., 1994) and other herpesviruses (Dargan et al., 2010; Tyler et al., 2007) *in vitro* is known to select mutants that have a growth advantage over wild‐type virus. The majority of viruses analysed in the present study were sequenced at passage 3 or 4 after their original isolation from the animals, to minimize the extent of adaptation, but it is possible that mutations occurred *in vitro*.

Analysis of the genome sequences derived in the present study identified four mutations that would truncate the cognate proteins. The strain Kent/43/1994 had a mutation in ORF63 (ICP0). Changes in a different region of ORF63 have been observed in the cell culture‐adapted EHV‐1 strain Kentucky A, which has a deletion of residues 319–431 compared to strain Ab4. Despite this, ICP0 in Kentucky A retains its activity (Bowles, Holden, Zhao, & O'Callaghan, 1997), even though mutational studies of HSV‐1 ICP0 have indicated that the *C*‐terminus can have an effect on protein intranuclear localization (Everett, 1988). The mutation in strain Buckinghamshire/9/93 ORF34 (protein V32) disrupts the *C*‐terminal part of the protein. The role of this protein in infection is not clear, although it is known that it is degraded via its *N* terminus through the ubiquitin proteosome pathway, and that deletion mutants lacking the whole ORF have a significant growth defect in PBMCs at early times post‐infection (Said, Damiani, & Osterrieder, 2014). The mutation in strain Cambridgeshire/96/2013 ORF14 (VP11/12) would truncate the protein by 124 residues. In HSV‐1, VP11/12 undergoes tyrosine phosphorylation in NK and T cells, but its biological function is unclear (Zahariadis et al., 2008). The strain Suffolk/45/2013 ORF11 was truncated by 99 residues. There was no evidence of the non‐deleted virus in the sequence data for this strain. The ORF11 protein (VP22) is highly conserved among alphaherpesviruses. EHV‐1 VP22 is required for efficient growth in cell culture, but is not essential for pathogenicity in the hamster model (Okada, Izume, Ohya, & Fukushi, 2015). The *C*‐terminal 89 residues of HSV‐1 VP22 contain a signal for cytoplasmic localization, and the *C*‐terminal 128 residues are required for chromatin‐binding activity (Martin, O'Hare, McLauchlan, & Elliott, 2002). In bovine herpesvirus 1 (BHV‐1) infection, full‐length VP22 has been shown to localize to the cell nucleus and interact with histones, and *C*‐terminally truncated forms have been shown to localize exclusively in the cytoplasm (Liang et al., 1995). Two of these mutations were due to deletions occurring within homopolymer tracts, which may suggest polymerase slippage had a role in protein evolution at these sites.

In conclusion, the present study describes the sequences of 78 EHV‐1 strains, most of which were isolated in the UK. Phylogenetic analysis of U_L_ identified up to 13 circulating viral clades differing from each other mainly by individual nucleotide variations spread throughout the genome. We identified a polymorphism in ORF30 that may be linked to neurological outbreaks and warrants further study. Moreover, the data suggest that intraspecies and interspecies recombinations of equine alphaherpesviruses have occurred. The extensive sequence information obtained adds considerable depth to that reported previously for EHV‐1, bringing the number of substantially complete genome sequences to over 100, thus helping to provide a comprehensive resource for fundamental and applied studies of EHV‐1.
